# Are Mercurials Anti-Syphilitics

**Published:** 1857-04

**Authors:** 


*Are Mercurials Anti-Syphilitics. Iodine and its Compounds.*—Such is the title of a pamphlet by C. H. Cleaveland, M. D., the leading mind among the eclectics of Cincinnati. As might be expected from the sectarian character of this writer, he takes the ultra anti-mercurial side ef the discussion. A good end is here gained, for his antipathy to mercury, while it leads him to decry unfairly its virtues, and exaggerate its dangers, at the same time induces a careful search for other remedies which may take its place and, perhaps, excel it in efficiency.

Some of his abstracts of the opinions of others, are included in the following extracts:

"Under those circumstances the majority of practitioners, doubtless resort to the *iodide of potassium*, and with good reason. But it may well be asked, if the iodide of potassium may be relied on in those cases where the whole system is seriously affected with the poison, or where the powers of the system are so far reduced that the mercurials would prove dangerous, why may we not also rely on this agent, where the amount of the poison is less, and the vitality more vigorous, and the system in a more normal state.

"Ricord has repeatedly said, that as the virus acts upon the system, in what he calls the *secondary*, and especially the *tertiary* forms, 'no remedy appears to be so uniformly and so potentially useful as the hydriodate of potash.' In almost all cases of syphilis affecting the periosteum and bone, he trusts to the iodide of potassium. For the *secondary* symptoms, M. Ricord sometimes uses the *proto-ioduret of iron.*

"In numerous instances where the ordinary methods of treatment had been ineffectually tried, the constitution seemed to rally quickly under the use of this preparation of steel, and the local disease at the same time began to assume a healthy action. 'Repeatedly have we seen,' says M. Ricord, old extensive ulcers of the throat, which had long resisted all the usual topical applications and internal medicines, rapidly change their aspect under the influence of the *proto-ioduret*, and within a very short time, heal and cicatrize. In many patients affected with caries of the bones of the face, cranium, and extremities, the process of separation of the diseased from the healthy parts has been surprisingly quickened, and the general health has at the same time been materially improved by its use.'

"Another set of symptoms, which has been much benefited by the use of the proto-ioduret, deserves to be especially noticed. We allude to old tedious discharges from the vagina and urethra, occurring in persons of enfeebled lymphatic constitutions. The dose which M. Ricord recommends, is six grs. during twenty-four hours at first; this is to be gradually increased to half a drachm or two scruples. Besides the internal administration of the ioduret, its external use, as an injection, has been extensively adopted by M. Ricord, in cases of obstinate gleet. The strength of the solution is usually half a drachm to eight ounces of water: in a few cases the quantity of the ioduret has been raised to two drachms.

"M. Baumes, head surgeon of the hospital at Lyons, has used this ferruginous preparation with most satisfactury results in the treatment of old and obstinate syphilitic ulcers, especially when the state of the patient was at the same time feeble and scrofulous. He administered it in the form of pills with Thebaic extract, increasing the dose of the ioduret from two or three to twelve or twenty grains in the course of twenty-four hours. Along with the cicatrization of the sores, the improvement of the general health was most remarkable; the appetite improved, the muscular strength increased, and the complexion acquired the florid hue of vigorous health. The salt, no doubt, is taken into the circulation, and acts on the blood itself, as well as on capillary vessels in every part of the body."

